# Detailed Sub-study Analysis of the SECRAB Trial: Quality of Life, Cosmesis and Chemotherapy Dose Intensity

**DOI:** 10.1016/j.clon.2023.03.007

**Published:** 2023-06

**Authors:** I.N. Fernando, S. Lax, S.J. Bowden, I. Ahmed, J.H. Steven, M. Churn, A.M. Brunt, R.K. Agrawal, P. Canney, A. Stevens, D.W. Rea

**Affiliations:** ∗ Cancer Centre, Queen Elizabeth Hospital, Birmingham, UK; † Cancer Research UK Clinical Trials Unit, University of Birmingham, Birmingham, UK; ‡ Clinical Oncology, Worcestershire Royal Hospital, Worcester, UK; § Cancer Centre, Royal Stoke University Hospital, Stoke on Trent, UK; ¶ Keele University, Keele, UK; || The Shrewsbury and Telford NHS Trust, Shrewsbury, UK; ∗∗ Beatson West of Scotland Cancer Centre, Glasgow, UK

**Keywords:** Breast cancer, chemotherapy, clinical trial, cosmesis, dose intensity, quality of life, radiotherapy, secondary outcomes, SECRAB

## Abstract

**Aims:**

SECRAB was a prospective, open-label, multicentre, randomised phase III trial comparing synchronous to sequential chemoradiotherapy (CRT). Conducted in 48 UK centres, it recruited 2297 patients (1150 synchronous and 1146 sequential) between 2 July 1998 and 25 March 2004. SECRAB reported a positive therapeutic benefit of using adjuvant synchronous CRT in the management of breast cancer; 10-year local recurrence rates reduced from 7.1% to 4.6% (*P* = 0.012). The greatest benefit was seen in patients treated with anthracycline–cyclophosphamide, methotrexate, 5-fluorouracil (CMF) rather than CMF. The aim of its sub-studies reported here was to assess whether quality of life (QoL), cosmesis or chemotherapy dose intensity differed between the two CRT regimens.

**Materials and methods:**

The QoL sub-study used EORTC QLQ-C30, EORTC QLQ-BR23 and the Women's Health Questionnaire. Cosmesis was assessed: (i) by the treating clinician, (ii) by a validated independent consensus scoring method and (iii) from the patients' perspective by analysing four cosmesis-related QoL questions within the QLQ-BR23. Chemotherapy doses were captured from pharmacy records. The sub-studies were not formally powered; rather, the aim was that at least 300 patients (150 in each arm) were recruited and differences in QoL, cosmesis and dose intensity of chemotherapy assessed. The analysis, therefore, is exploratory in nature.

**Results:**

No differences were observed in the change from baseline in QoL between the two arms assessed up to 2 years post-surgery (Global Health Status: –0.05; 95% confidence interval –2.16, 2.06; *P* = 0.963). No differences in cosmesis were observed (via independent and patient assessment) up to 5 years post-surgery. The percentage of patients receiving the optimal course-delivered dose intensity (≥85%) was not significantly different between the arms (synchronous 88% versus sequential 90%; *P* = 0.503).

**Conclusions:**

Synchronous CRT is tolerable, deliverable and significantly more effective than sequential, with no serious disadvantages identified when assessing 2-year QoL or 5-year cosmetic differences.

## Highlights

• SECRAB assessed synchronous or sequential CRT in breast cancer.
• Synchronous CRT showed positive therapeutic benefit; sub-studies are reported here.
• Quality of life was similar in patients receiving synchronous or sequential CRT.
• Cosmesis was similar in patients receiving synchronous or sequential CRT.
• Chemotherapy dose intensity was comparable between the two CRT regimens.

## Introduction

The standard treatment for operable breast cancer is surgery. Some patients then also require adjuvant chemotherapy and radiotherapy. The largest trial to date, SECRAB, opened in 1998 when a wide range of chemoradiotherapy (CRT) schedules was used, with no evidence to favour sequential or synchronous protocols. The primary objective of this randomised phase III trial was to investigate whether local control could be improved by synchronous delivery of adjuvant chemotherapy and radiotherapy, not delaying the administration of either modality. This pragmatic trial recruited 2297 patients (1150 assigned to the synchronous arm and 1146 to the sequential arm) from 48 centres in the UK between 2 July 1998 and 25 March 2004. After 10 years of follow-up, SECRAB concluded that synchronous CRT significantly improves locoregional recurrence rates when compared with sequential chemotherapy followed by radiotherapy; 4.6% versus 7.1%, respectively (hazard ratio 0.62; 95% confidence interval 0.43, 0.90; *P* = 0.012) [1]. The greatest benefit of synchronous CRT was in patients treated with anthracycline–cyclophosphamide, methotrexate, 5-fluorouracil (CMF), even though these patients would have had a greater delay in starting radiotherapy compared with those patients treated with CMF. There was an increase in acute skin toxicity in patients treated with synchronous treatment, predominantly in those treated with >3 week fractionation, but no significant difference in dose reduction of >20% in chemotherapy, and no differences in the late effects of radiotherapy [1].

Additional secondary endpoints of SECRAB included whether synchronous CRT could be given safely without enhancement of acute or late toxicity, or a significant reduction in chemotherapy dose intensity, as it has previously been acknowledged that the doses of chemotherapy received are rarely 100% of the projected dose due to toxicity [2,3]. Therefore, here we report the detailed analyses of these quality of life (QoL), cosmesis and chemotherapy dose-intensity sub-studies.

## Materials and Methods

### Study Design

SECRAB was a large randomised, phase III trial comparing sequential chemotherapy followed by radiotherapy with synchronous radiotherapy given concurrently or as a sandwich with chemotherapy [1]. Permitted chemotherapy regimens were CMF (intravenous or oral; six cycles), four cycles of anthracycline followed by four cycles of CMF or mitomycin-C, mitoxantrone and methotrexate with six widely used standard radiotherapy schedules permitted, ranging from 15 to 25 daily fractions with or without subsequent boost doses [1]. The secondary endpoints of QoL, toxicity, cosmesis and dose intensity were assessed in sub-studies of ≥300 patients. Only centres electing to study these questions and with the resources to provide more detailed study data participated in the sub-studies. Patients who had already started chemotherapy were not eligible to enter the patient-reported sub-studies, as baseline data before the start of adjuvant treatment were required.

The protocol and subsequent amendments were approved by the West Midlands Multi-centre Research Ethics Committee and by the research and development department at each centre. The trial was carried out in accordance with The Code of Ethics of the World Medical Association (Declaration of Helsinki). All patients provided informed consent.

### Patients

Patients with histologically confirmed, invasive, early stage breast cancer with no evidence of metastatic disease were eligible for this study. Patients were required to have complete macroscopic excision of their tumour by mastectomy or breast-conserving surgery. Full eligibility criteria are listed in the trial publication [1].

### Quality of Life

QoL booklets comprised the EORTC QLQ-C30 [4], the EORTC QLQ-BR23 [5] and the Women's Health Questionnaire [6]. Patient diary sheets were completed by participating patients at four specified time points during their treatment; at baseline (prior to any chemotherapy), on completion of both chemotherapy and radiotherapy and at 1 and 2 years post-surgery.

Centres that participated in the QoL sub-study are listed in Supplementary Appendix 1.

### Cosmesis

Cosmetic assessment was from three perspectives: the treating clinician, via independent consensus using photographs and the patient.

The treating clinician assessed cosmesis following wide local excision (WLE) and telangiectasia following mastectomy or WLE. Both were assessed at the end of adjuvant treatment and at 1, 2 and 5 years post-surgery and categorised on a four-point scale from poor to excellent [7,8]. Acute skin reaction to radiotherapy was assessed within 2 weeks of the completion of radiotherapy and at the first follow-up visit following the end of all adjuvant treatment, and categorised as none, mild, moderate or severe [9]. This system shows simplified equivalence to the toxicity criteria of the Radiation Therapy Oncology Group in which moderate skin reaction would be completely healed within 4 weeks and severe skin toxicity would not be healed by 4 weeks [10].

For the independent assessment after WLE or mastectomy, photographs were taken at baseline, 1, 2 and 5 years post-surgery. As implemented in the Standardisation of Radiotherapy (START) trial that was run in parallel to SECRAB [11], changes in breast appearance in these photographs were scored by three observers (AMB, MC and JHS) blind to patient identity, treatment allocation and year of follow-up, and a final agreed score reached by consensus with random blind re-assessment of 10% of scores [12]. Changes in breast appearance overtime were scored as minimal (identical or minor differences), mild (clearly different) or marked (very different).

Although an indirect measure, importantly, the patient's own assessment was also analysed at baseline and at each patient's final assessment (either 1, 2 or 5 years after surgery, whichever was their last available time point) using their responses to specific QoL-related questions from the EORTC QLQ-BR23 questionnaire: Question 9 – Have you felt physically less attractive as a result if your disease or treatment?; Question 10 – Have you been feeling less feminine as a result of your breast cancer treatment?; Question 11 – Did you find it difficult to look at yourself naked?; and Question 12 – Have you been dissatisfied with your body?.

Centres that participated in the cosmesis sub-study are listed in Supplementary Appendix 2.

### Chemotherapy Dose Intensity

For each cycle, data were collected on chemotherapy doses and dates. These data were captured from pharmacy records. All records used were complete.

### Statistical Analysis

SECRAB was powered only to detect differences in local recurrences rates, the primary outcome. Consequently, the sub-studies were not formally powered, rather it was the aim to use at least 300 patients (150 in each arm) to compare differences in QoL, cosmesis and dose intensity of chemotherapy. The analysis, therefore, is exploratory in nature and no adjustments for multiple testing have been made. In addition, the numbers of patients per arm for each sub-study were not randomised and were instead dependent only on the number of patients who agreed to take part in them.

#### Quality of Life

All three questionnaires were analysed using multi-level mixed effects models, where repeated measurements from baseline through to 2 years post-surgery were analysed as random effects with stratification factors and radiotherapy schedule forced into the model as fixed effects. Trial stratification factors included centre, axillary surgery, chemotherapy regimen and inclusion of radiotherapy boost. In addition, treatment and questionnaire form number were included as interaction terms, i.e. additional variables upon which outcomes are dependent. The multi-level mixed effects model analysis takes into consideration the type and patterns of missing data. All raw data (mean with standard deviations) are shown graphically, with adjusted point estimates and confidence intervals presented within the text and tables.

#### Cosmesis

For cosmesis following WLE and telangiectasia following mastectomy or WLE, data were compared to baseline from the first follow-up visit to 5 years post-surgery. Data were classified as improved, stable or worsened, and a Fisher's exact test used to assess the differences. Odds ratios of moderate/poor versus excellent/good assessment comparing synchronous to sequential CRT treatments were analysed using each patient's last available assessment compared to baseline. For acute skin reactions, odds ratios of moderate/severe versus none/mild comparing synchronous to sequential CRT treatments were analysed at the two time points. For the independent review, odds ratios of marked/mild versus minimal comparing synchronous to sequential CRT treatments were analysed using each patient's last available assessment compared to baseline. For each cosmesis-related QoL question, data from the patient's last available assessment were classified as improved, stable or worsened and a Fisher's exact test used to assess the differences.

#### Chemotherapy

Course-delivered dose intensity (CDDI) was calculated as follows: (i) a per drug dose intensity (administered dose per day divided by the planned mg m^−2^ day^−1^); (ii) a per cycle dose intensity (averaging all drug dose intensities planned for that cycle); and (iii) a per patient CDDI was calculated (averaging the above over all planned cycles). Patients with calculable CDDI were compared using Fisher's exact test.

## Results

### Quality of Life

In total, 748 (33%) patients completed at least one QoL form; 381 (33%) on the synchronous arm and 367 (32%) on the sequential arm. Sixteen patients had baseline assessments missing, 76 only had baseline assessments and 85 had commenced chemotherapy prior to their baseline assessment; all were excluded. Five hundred and seventy-one patients were evaluable and included in the analysis.

When compared to the patient characteristics of all patients randomised within the SECRAB trial [1], significant differences were observed between several categories, including whether the patient underwent axillary node dissection or received a radiotherapy boost (Table 1). In addition, whether patients had ovarian ablation or received tamoxifen differed between the sub-study and parent trial (Table 1). The distribution of patient characteristics for patients participating in the QoL sub-study were balanced across the two arms (see Supplementary Appendix 3 and Table 1).

**Table 1:** Differences in treatments and baseline characteristics of patients included in the SECRAB quality of life sub-study compared to the parent trial

	Quality of life sub-study			Difference to full trial population		
	Synchronous	Sequential	Total	*P* value	Synchronous	Sequential
	*n* = 291 (%)	*n* = 280 (%)	*n* = 571 (%)		*n* = 1150 (%)	*n* = 1146 (%)
Axillary node dissection performed				<0.001		
No	111 (38)	104 (37)	215 (38)		292 (25)	585 (26)
Yes	180 (62)	176 (63)	356 (62)		858 (75)	853 (74)
Radiotherapy boost				<0.001		
No	234 (80)	218 (78)	452 (79)		803 (70)	762 (67)
Yes	57 (20)	62 (22)	119 (21)		340 (30)	360 (32)
Radiotherapy not given	–	–	–		6 (0)	23 (2)
Missing	–	–	–		1 (0)	1 (0)
Endocrine therapy or ovarian ablation				0.017		
No	265 (91)	251 (90)	516 (90)		1077 (94)	1063 (93)
Yes	25 (9)	28 (10)	53 (9)		64 (6)	75 (7)
Unknown	1 (0)	1 (0)	2 (0)		9 (1)	8 (1)
Tamoxifen				0.001		
No	84 (29)	78 (28)	162 (28)		363 (32)	353 (31)
Yes, with chemotherapy	111 (38)	90 (32)	201 (35)		319 (28)	295 (26)
Yes, after chemotherapy	95 (33)	111 (40)	206 (36)		454 (40)	490 (43)
Unknown	1 (0)	1 (0)	2 (0)		14 (1)	8 (1)

Note: Percentages may not total 100 due to rounding.

Across the three questionnaires contained within the QoL booklet, 3023/6429 (47%) questionnaires were fully completed; 1595/3294 (48%) in the synchronous arm and 1428/3135 (46%) in the sequential arm. Two hundred and three (3%) questionnaires had five or more unknown responses; 99 (3%) in the synchronous arm and 104 (3%) in the sequential arm.

**Fig 1:**
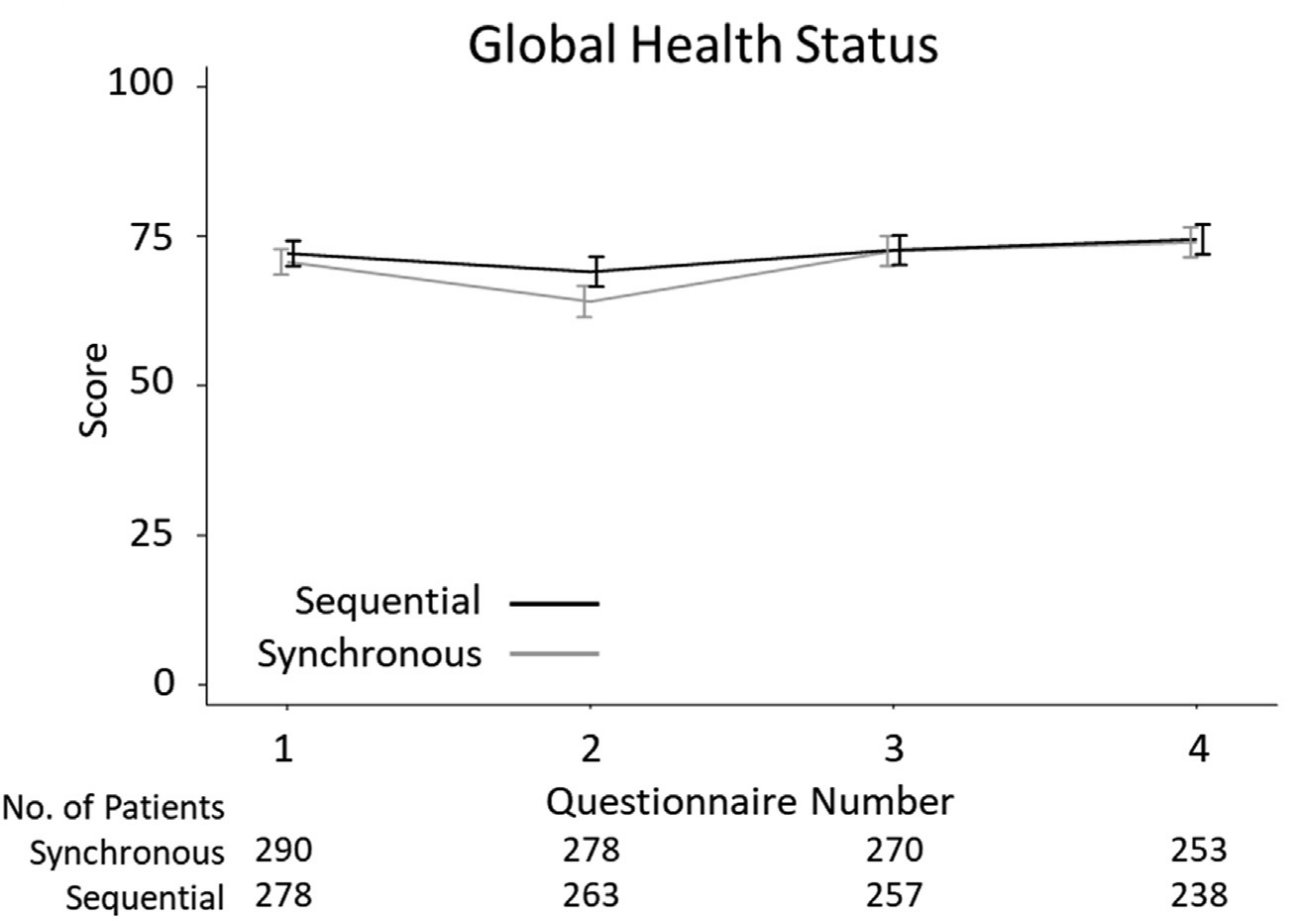
Global health status of patients within the SECRAB quality of life sub-study. The unadjusted mean and standard deviation of changes in patients' global health status receiving synchronous or sequential chemoradiotherapy as assessed by EORTC QLQ-C30 are shown. Questionnaire numbers: 1 – baseline assessment prior to chemotherapy; 2 – on completion of both chemotherapy and radiotherapy; 3–1 year after surgery; 4–2 years after surgery.

As assessed by the EORTC QLQ-C30, no significant difference was observed between the two arms over the duration of the 2-year sub-study in global health status (–0.05; 95% confidence interval –2.16, 2.06; *P* = 0.963; Figure 1) nor within any of the component scales or measures (see Supplementary Appendix 4).

Following analysis via multi-level mixed effects, at the completion of both chemotherapy and radiotherapy (questionnaire 2) compared with baseline, patients' QoL was significantly different between the two arms in a subset of the measures. These measures included reduced physical functioning (–5.76; 95% confidence interval –8.89, –2.53; *P* < 0.001), reduced role functioning (–4.75; 95% confidence interval –9.24, –0.26; *P* = 0.038), increased dyspnoea (5.98; 95% confidence interval 2.04, 9.91; *P* = 0.003) and increased insomnia (5.96; 95% confidence interval 0.67, 1.24; *P* = 0.027) in those patients who received synchronous CRT compared to sequential. These differences were not present at later time points.

No differences were observed between the two arms in the emotional functioning, cognitive functioning, social functioning, fatigue, nausea and vomiting, pain, appetite loss, constipation or diarrhoea measures in any of the questionnaires compared to baseline (see Supplementary Appendix 4).

At 1 year post-treatment, compared to baseline the only measure that showed a significant difference was financial difficulties; patients receiving synchronous CRT perceived their treatment to have caused them fewer financial difficulties (–6.21; 95% confidence interval –10.78, –1.64; *P* = 0.008). In addition, this was found to be only in those patients who received a radiotherapy schedule of >3 weeks (4.40; 95% confidence interval 1.06, 7.74; *P* = 0.010).

**Fig 2:**
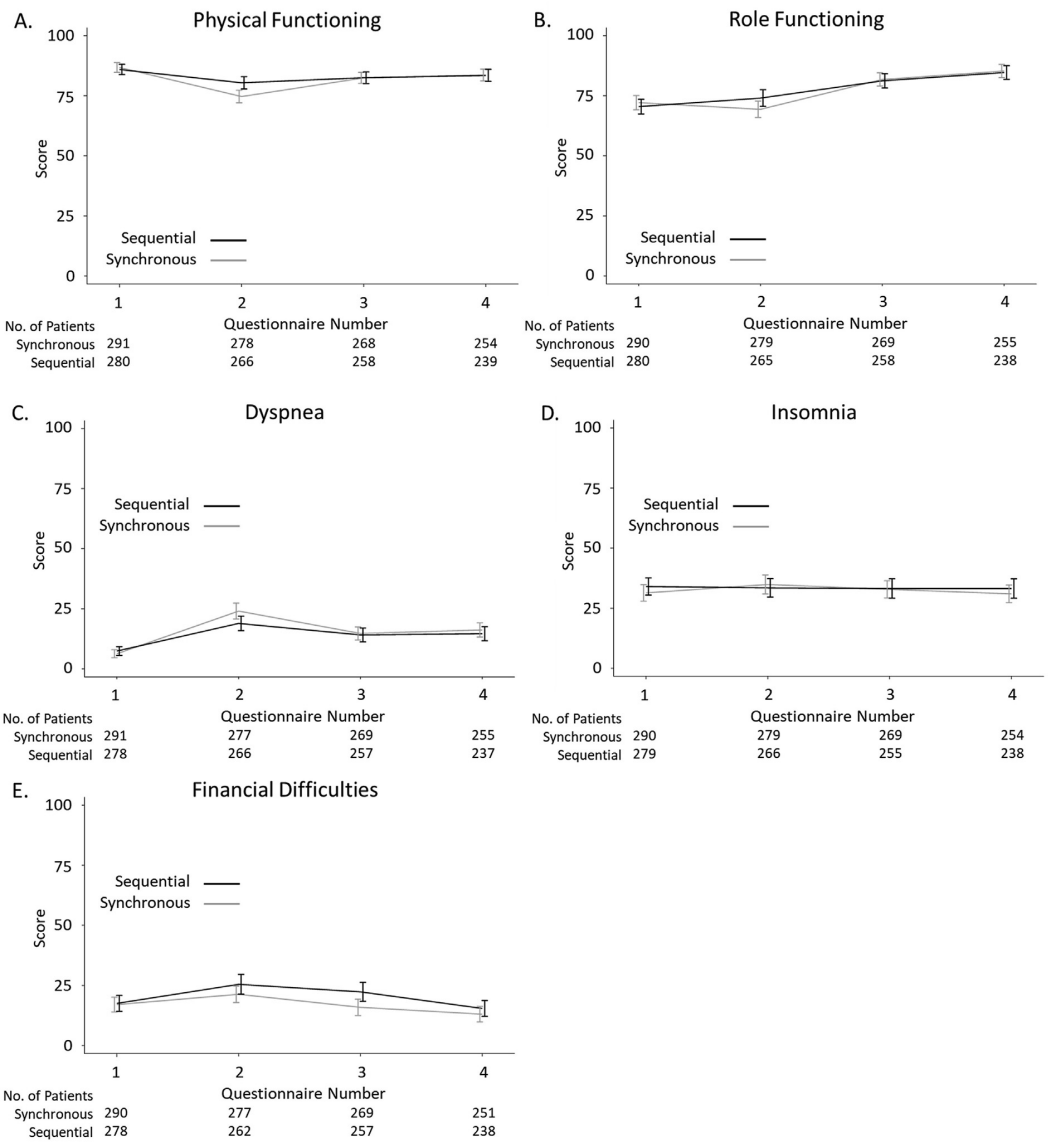
EORTC QLQ-C30 sub-scale differences within the SECRAB quality of life sub-study. The unadjusted mean and standard deviations of changes within the EORTC QLQ-C30 sub-scales in patients receiving synchronous or sequential chemoradiotherapy are shown. Questionnaire numbers: 1 – baseline assessment prior to chemotherapy; 2 – on completion of both chemotherapy and radiotherapy; 3–1 year after surgery; 4–2 years after surgery.

Figure 2 shows the unadjusted comparisons for the QoL functional sub-scales that were found to be significantly different following mixed-method analyses.

There were no significant differences observed between patients receiving synchronous or sequential CRT across all scales and measures within the EORTC-BR23 during the 2-year follow-up (unadjusted data; see Supplementary Appendix 5). When each questionnaire was assessed compared to baseline (adjusted data), breast symptoms were lower at completion of all radiotherapy and chemotherapy in those patients receiving synchronous regimens (–5.10; 95% confidence interval –8.07, –2.13; *P* = 0.001) and at 1 year post-treatment sexual functioning was higher (4.62; 95% confidence interval 0.62, 8.61; *P* = 0.024) with lower systemic therapy side-effects (–1.00; 95% confidence interval –1.69, –0.31; *P* = 0.004) in those patients who received synchronous CRT compared to sequential and compared to baseline. The model was not able to analyse the future perspective item.

No statistical differences were observed in the mental and physical health sub-scales of the Women's Health Questionnaire, when comparing patients who received synchronous CRT with those who received sequential treatment regimens during the 2-year post-treatment follow-up period (see Supplementary Appendix 4).

### Cosmesis

Three hundred and eighty patients were enrolled into the cosmesis sub-study; 324 of whom had at least a baseline assessment and were included in the clinician's assessment analysis. One hundred and eighty patients received synchronous CRT, with 144 receiving sequential. This imbalance was possibly due to the fact that most of the consultants enrolling into the cosmesis sub-study were from the West Midlands and tended to use anthracycline-CMF, with 3-weekly radiotherapy. Although numbers of patients per arm differed, baseline characteristics were balanced between the arms (see Supplementary Appendix 6 and Table 2).

**Table 2:** Differences in treatments and baseline characteristics of patients included in the SECRAB cosmesis sub-study compared to the parent trial

	Cosmesis sub-study			Difference to full trial population		
	Synchronous	Sequential	Total	*P* value	Synchronous	Sequential
	*n* = 180 (%)	*n* = 144 (%)	*n* = 324 (%)		*n* = 1150 (%)	*n* = 1146 (%)
Axillary node dissection performed				<0.001		
No	95 (53)	75 (52)	170 (52)		292 (25)	293 (26)
Yes	85 (47)	69 (48)	154 (48)		858 (75)	853 (74)
Chemotherapy regimen intent				<0.001		
CMF	64 (36)	58 (40)	122 (38)		617 (54)	527 (55)
Anthracycline–CMF	113 (63)	86 (60)	199 (6)		525 (46)	516 (45)
MMM	3 (2)	–	3 (1)		6 (0)	3 (0)
Radiotherapy schedule intent				<0.001		
3-weekly	152 (84)	133 (78)	265 (82)		880 (76)	855 (75)
>3-weekly	28 (16)	31 (22)	59 (18)		260 (23)	263 (23)
Not given/missing	–	–	–		10 (1)	28 (2)
Radiotherapy boost				<0.001		
No	135 (75)	98 (68)	233 (72)		803 (70)	762 (67)
Yes	45 (25)	46 (32)	91 (28)		340 (30)	360 (32)
Radiotherapy not given	–	–	–		6 (0)	23 (2)
Missing	–	–	–		1 (0)	1 (0)
ER Status				0.006		
Negative	58 (32)	51 (35)	109 (34)		401 (35)	387 (34)
Positive	120 (67)	93 (65)	213 (66)		703 (61)	724 (63)
Unknown	2 (1)	–	2 (1)		46 (4)	35 (3)
PgR Status				<0.001		
Negative	47 (26)	45 (31)	92 (28)		250 (22)	255 (22)
Positive	60 (33)	39 (27)	99 (31)		265 (23)	258 (23)
Unknown	73 (41)	60 (42)	133 (41)		635 (55)	623 (55)
HER2 Status				0.007		
Negative	24 (13)	22 (15)	46 (14)		104 (9)	102 (11)
Positive	11 (6)	8 (6)	19 (6)		39 (3)	44 (4)
Unknown	145 (81)	114 (79)	259 (80)		1007 (88)	980 (85)
Endocrine therapy Ovarian ablation				<0.001		
No	157 (87)	122 (85)	279 (86)		1077 (94)	1063 (93)
Yes	22 (12)	22 (15)	44 (15)		64 (6)	75 (7)
Unknown	1 (1)	–	1 (0)		9 (1)	8 (1)
Tamoxifen				0.001		
No	61 (34)	48 (33)	109 (34)		363 (32)	353 (31)
Yes, with chemotherapy	72 (40)	57 (40)	129 (40)		319 (28)	295 (26)
Yes, after chemotherapy	47 (26)	38 (26)	85 (26)		454 (40)	490 (43)
Unknown	–	1 (1)	1 (0)		14 (1)	8 (1)

CMF, cyclophosphamide, methotrexate and 5-fluorouracil; ER, oestrogen receptor; HER2, human epidermal growth factor receptor 2; MMM, mitomycin-C, mitoxantrone and methotrexate; PgR, progesterone receptor.

Note: Percentages may not total 100 due to rounding.

When compared with the patient characteristics of all patients randomised within the SECRAB trial [1], significant differences were observed between several categories, including whether the patient underwent 3-weekly fractionation, axillary dissection, their chemotherapy regimen intent and whether they received a radiotherapy boost (Table 2). In addition, whether patients had ovarian ablation or received tamoxifen and the patients' oestrogen receptor, progesterone receptor and human epidermal growth factor receptor 2 statuses were all significantly different between the sub-study and parent trial (Table 2). Of the 324 included in the cosmesis analysis, 57 remained on study and had data at all six assessments, the remaining 267 had incomplete data.

No differences in acute skin reactions were observed between synchronous and sequential CRT treatment at the initial assessment, or at the first follow-up visit following the end of all adjuvant treatment within this sub-study (Table 3).

**Table 3:** Analysis of the SECRAB cosmesis sub-study

Assessments compared to baseline	Synchronous *n* (%)	Sequential *n* (%)	Overall *n* (%)	*P* value	Odds ratio (95% confidence interval)
**Cosmesis following WLE**					
First follow-up visit∗					
Improved	14 (21)	18 (32)	32 (26)		
Stable	36 (54)	2 (48)	63 (51)	0.356	–
Worsened	17 (25)	11 (20)	28 (23)		
Total	67	56	123		
1-year follow-up					
Improved	16 (24)	18 (35)	34 (29)		
Stable	30 (45)	24 (47)	54 (46)	0.217	–
Worsened	20 (30)	9 (18)	29 (25)		
Total	66	51	117		
2-year follow-up					
Improved	18 (31)	21 (36)	39 (33)		
Stable	25 (42)	25 (43)	50 (43)	0.706	–
Worsened	16 (27)	12 (21)	28 (24)		
Total	59	58	117		
5-year follow-up					
Improved	6 (20)	15 (52)	21 (36)		
Stable	16 (53)	12 (41)	28 (47)	0.019	–
Worsened	8 (27)	2 (7)	10 (17)		
Total	30	29	59		
Last available	79	58	147	0.067	2.13 (0.95–4.77)
**Telangiectasia following mastectomy or WLE**					
First follow-up visit∗					
Improved	4 (3)	0 (0)	4 (2)		
Stable	85 (66)	61 (69)	146 (67)	0.312	–
Worsened	39 (30)	28 (31)	67 (31)		
Total	128	89	217		
1-year follow-up					
Improved	5 (4)	2 (3)	7 (4)		
Stable	80 (66)	56 (71)	136 (68)	0.759	–
Worsened	36 (30)	21 (27)	57 (29)		
Total	121	79	200		
2-year follow-up					
Improved	3 (3)	1 (1)	4 (2)		
Stable	60 (57)	51 (64)	111 (60)	0.588	–
Worsened	43 (41)	28 (35)	71 (38)		
Total	106	80	86		
5-year follow-up					
Improved	1 (2)	0 (0)	1 (1)		
Stable	29 (63)	28 (74)	57 (68)	0.548	–
Worsened	16 (35)	10 (26)	26 (31)		
Total	46	38	84		
Last available	160	18	278	0.955	1.03 (0.32–3.34)
**Acute skin reaction to radiotherapy**					
End of treatment†					
None	8 (6)	9 (8)	17 (7)		
Mild	103 (72)	80 (72)	183 (72)	0.551	1.20 (0.66–2.21)
Moderate	29 (20)	21 (19)	50 (20)		
Severe	4 (3)	1 (1)	5 (2)		
Total	144	111	255		
First follow-up visit∗					
None	66 (48)	39 (39)	105 (44)		
Mild	61 (44)	51 (51)	112 (47)	0.587	0.78 (0.32–1.91)
Moderate	8 (6)	6 (6)	14 (6)		
Severe	3 (2)	4 (4)	7 (3)		
Total	138	100	238		
**Independent assessment after WLE**					
Minimal	52 (72)	46 (73)	98 (73)		
Mild	19 (26)	17 (27)	36 (27)	0.918	1.04 (0.49–2.22)
Moderate	1 (1)	0 (0)	1 (1)		
Total	72	63	135		
**Last available patient's assessment via EORTC QLQ-BR23**					
Question 9					
Improved	16 (10)	10 (8)	26 (9)		
Stable	125 (77)	110 (83)	235 (80)	0.393	–
Worsened	22 (13)	12 (9)	34 (12)		
Total	163	132	295		
Question 10					
Improved	11 (7)	11 (8)	22 (7)		
Stable	132 (81)	107 (80)	239 (81)	0.860	–
Worsened	20 (12)	15 (11)	35 (12)		
Total	163	133	296		
Question 11					
Improved	14 (9)	7 (5)	21 (7)		
Stable	126 (78)	117 (87)	243 (82)	0.105	–
Worsened	22 (14)	10 (7)	32 (11)		
Total	162	134	296		
Question 12					
Improved	10 (6)	7 (5)	17 (6)		
Stable	126 (78)	107 (80)	233 (79)	0.902	–
Worsened	25 (16)	19 (14)	44 (15)		
Total	161	133	294		

WLE, wide local excision.

Note: Percentages may not total 100 due to rounding.

∗ At the end of all adjuvant treatment (usually 6–12 weeks).

† Within 1 week of completing radiotherapy.

There were also no differences in late toxicity observed at endpoints between sequential and concurrent treatment (as assessed via the treating clinician, via independent consensus using photographs and the patient). The only exception was the physician assessment at 5 years; here, a statistically significant improvement was observed in cosmesis following WLE in those patients who received sequential CRT compared to synchronous (Table 3). However, it is noted that there were fewer patients assessed at this late time point compared with previous assessments.

### Chemotherapy Dose Intensity

To check that concurrent treatment did not diminish chemotherapy dose intensity received, it was calculated for 831 (36%) patients having 5740 cycles of chemotherapy (2913 cycles from 421 patients on the synchronous arm and 2827 cycles from 410 patients on the sequential arm). Overall dose intensity for all drugs combined across all cycles are reported in Table 4. No significant differences were observed between any of the data presented (synchronous 88% versus sequential 90%; *P* = 0.503).

**Table 4:** Overall dose intensity for all chemotherapy regimens used during SECRAB

Chemotherapy regimen	Synchronous *n* = 421 (%)	Sequential *n* = 410 (%)	Total *n* = 831
All chemotherapy regimens			
≤85% CDDI	49 (12)	41 (10)	90 (11)
≥85% CDDI	372 (88)	369 (90)	741 (89)
CMF regimens			
CMF ‘classical’			
≤85% CDDI	18 (14)	14 (11)	32 (13)
≥85% CDDI	107 (86)	117 (89)	224 (88)
CMF ‘classical’ i.v.			
≤85% CDDI	7 (15)	9 (18)	16 (17)
≥85% CDDI	39 (85)	40 (82)	79 (83)
CMF (6–8) i.v. 3-weekly (Scottish Breast Group Schedule)			
≤85% CDDI	2 (8)	1 (4)	3 (6)
≥85% CDDI	24 (92)	23 (96)	47 (94)
Anthracycline-containing regimens			
Epirubicin + ‘classical’ CMF			
≤85% CDDI	21 (10)	16 (8)	37 (9)
≥85% CDDI	186 (90)	182 (92)	368 (91)
3-weekly epirubicin/CMF (Scottish Breast Group Schedule)			
≤85% CDDI	0 (0)	0 (0)	0 (0)
≥85% CDDI	7 (100)	3 (100)	10 (100)
Bonadonna regimen			
≤85% CDDI	0 (0)	0 (0)	0 (0)
≥85% CDDI	4 (100)	2 (100)	6 (100)
Mitomycin-C, mitoxantrone and methotrexate			
≤85% CDDI	1 (17)	1 (33)	2 (22)
≥85% CDDI	5 (83)	2 (67)	7 (78)

CDDI, course-delivered dose intensity; CMF, cyclophosphamide, methotrexate, 5-fluorouracil; i.v., intravenous.

## Discussion

The results of the SECRAB trial have been published in details elsewhere [1], but, in summary, show that for any patient having anthracycline–CMF, radiotherapy given synchronously between the first and second cycles of CMF using a 3-week fractionation had a significantly lower locoregional recurrence rate.

SECRAB was powered only to detect differences in local recurrences rates, the primary outcome. Consequently, the sub-studies were not formally powered, with analyses presented here exploratory in nature. In addition, patients in the QoL and cosmetic sub-studies were from selected centres and were not randomised. As a result, there were differences between those within these analyses and the main study in several factors, which have been discussed. Some of these imbalances may have arisen due to the fact that most of the consultants enrolling patients into the cosmesis study were from the West Midlands and tended to use anthracycline–CMF with 3-weekly radiotherapy. In addition, one hospital, which recruited heavily into the sub-studies, favoured sampling to the axilla if node positive rather than axillary node clearance followed by radiotherapy.

The baseline characteristics of the patients within the QoL sub-study were equally balanced between those who received synchronous versus sequential schedules. In addition, although a significant number of patients did not complete all QoL forms, this was equally balanced in the two arms of the study. As noted, significant differences were seen compared to the main study in terms of axillary node dissection performed, radiotherapy boost and endocrine therapy with tamoxifen and/or ovarian ablation.

No difference in global well-being between patients receiving synchronous or sequential CRT was observed. Minor differences were seen at the end of treatment in physical and role functioning, and dyspnoea, but these had all resolved by 12 months post-treatment. Of note, there was no additional pain, fatigue or insomnia from synchronous chemotherapy. There was, however, a benefit seen in breast symptoms at the end of all treatment in the synchronous arm, probably as radiotherapy had been given well before the end of chemotherapy. Also, both sexual function and financial difficulties favoured patients treated with synchronous schedules compared to sequential patients. The latter was possibly because patients could return to work after their last chemotherapy cycle in the synchronous group, whereas those in the sequential arm may need to delay a return to work until after the completion of radiotherapy, which may not even start until 4 weeks after chemotherapy.

Patients were also equally balanced in the cosmesis sub-study in terms of baseline characteristics, but again there were significant differences to the main study in terms of axillary node dissection performed, chemotherapy and radiotherapy intent, if a radiotherapy boost was given and hormone therapy.

No difference in acute toxicity was seen in the cosmesis sub-study. This is at variance to the main study, which had shown a significantly higher rate of moderate/severe toxicity in patients treated using synchronous treatment; this was particularly evident in patients treated with >3 weekly fractionation [1]. These differences may have been because there was a lower proportion of patients who received the 3-weekly fractionation in the cosmesis sub-study compared with the parent trial. This may have also contributed to the slightly lower rates of telangiectasia observed. Interestingly, a higher proportion of patients in the cosmesis sub-study received anthracycline-CMF, which is reassuring as this was the preferred group who seemed to benefit most in terms of locoregional control from synchronous treatment [1].

There was an apparent improvement in cosmetic outcome in the sequential arm compared to those treated with synchronous treatment at the 5-year follow-up. This may have been due to small numbers of patients at this time point, as at all other time points, and using only the last available assessment for each patient, no differences were detected. It is also noteworthy to mention that clinicians were aware whether patients had been given synchronous or sequential treatment so their assessment was not blinded. This is in contrast to the independent assessments, which were blinded to treatment allocation and no difference was observed. Finally, these data are also in agreement with those received from the patients' responses to specific QoL-related questions within the EORTC QLQ-BR23 questionnaire, which indicated no differences in cosmesis between the two treatment modalities.

Significant differences in late toxicity effects were seen in the ARCOSEIN study, which used a concurrent anthracycline (mitoxantrone) regimen [13]; patients were treated with 50 Gy in 25 fractions with rates of subcutaneous fibrosis, telangiectasia, skin pigmentation and breast atrophy significantly worse in the concurrent arm. Results from the phase III trial by Rouëssé *et al.* [14], which again used a mitoxantrone-containing regimen also showed worse acute skin toxicity and telangiectasia in patients who received concomitant radiotherapy. However, the 3-year global cosmetic evaluations by physicians and patients were not statistically different in the two arms. In addition, there was no significant difference in dose intensity of chemotherapy administration. Mitoxantrone-containing chemotherapy regimens are no longer used due to the increased rate of haematological malignancy, with CMF-containing chemotherapy regimens showing significantly less acute and late toxicity, as seen in our own study [1] and in the smaller study by Arcangeli *et al.* [15], which showed low levels of acute skin toxicity. Retrospective data have suggested that cosmetic results are worse with concurrent CMF and adjuvant radiotherapy [16]. However, this was using a 5-week radiotherapy schedule, whereas our results were predominantly using a 3-week fractionation, which may account for the difference in results as radiotherapy could be sandwiched between chemotherapy courses.

Taxanes were not used as standard treatment when SECRAB was set up and we have discussed previously in our main publication the dangers of using taxanes synchronously with radiotherapy in view of the increased risk of pneumonitis [17]. However, it would be feasible to use synchronous treatment with regimens such as epirubicin-docetaxel-CMF, where synchronous treatment is still possible with CMF but treatment is sequential to taxanes [18].

The median CDDI was high in SECRAB at 88% for synchronous regimens and 90% for sequential regimens (*P* = 0.503). These data confirm the deliverability of the synchronous treatment schedules with no significant effect on dose intensity of chemotherapy between the two arms.

In summary, the results of the SECRAB sub-studies show that there are no long-term QoL or cosmesis effects using synchronous CRT compared with sequential treatment regimens, and that synchronous CRT is deliverable. This supports the main SECRAB trial outcome, which demonstrated the benefit of using synchronous CRT in the management of breast cancer by reducing 10-year local recurrence rates.

## Clinical Trial Registration

ClinicalTrials.gov NCT00003893.

## Ethics Statement

The protocol and subsequent amendments were approved by the West Midlands Multi-centre Research Ethics Committee and by the research and development department at each centre.

## Data Availability

Participant data and the associated supporting documentation will be available within 6 months after the publication of this manuscript. Details of our data request process is available on the Cancer Research UK Clinical Trials Unit (CRCTU) website. Only scientifically sound proposals from appropriately qualified research groups will be considered for data sharing. The decision to release data will be made by the CRCTU Director's Committee, who will consider the scientific validity of the request, the qualifications and resources of the research group, the views of the Chief Investigator and the trial steering committee, consent arrangements, the practicality of anonymising the requested data and contractual obligations. A data sharing agreement will cover the terms and conditions of the release of trial data and will include publication requirements, authorship and acknowledgements and obligations for the responsible use of data. An anonymised encrypted dataset will be transferred directly using a secure method and in accordance with the University of Birmingham's IT guidance on encryption of data sets.

## Author Contributions

INF is the guarantor of integrity of the entire study. INF, SJB, MC, RKA, AMB, AS, PC and DWR were responsible for study concepts and design. INF, JHS, MC, RKA, AMB, AS, PC and DWR were responsible for the clinical studies. INF, SL, SJB and IA carried out the experimental studies/data analysis. IA carried out the statistical analysis. INF, SL, SJB and IA prepared the manuscript. INF, SL, SJB, IA, JHS, MC, RKA, AMB, AS, PC and DWR edited the manuscript.

## Funding

This work was supported by 10.13039/501100000289Cancer Research UK (CRUK/98/001). A small educational grant was provided by Pharmacia for collection of photographs used for the cosmesis assessments. The funders of the study had no role in study design, data collection, data analysis, data interpretation or writing of the report. The corresponding author had full access to all the data in the study. The corresponding author had final responsibility for the decision to submit for publication.

## Conflicts of Interest

I.N. Fernando and S.J. Bowden received grants from 10.13039/501100000289Cancer Research UK and Pharmacia pertaining to this research but have no other competing interests. All other authors declare no competing interests.
