# Supplementary material for: Detailed Sub-study Analysis of the SECRAB Trial: Quality of Life, Cosmesis and Chemotherapy Dose Intensity
Source: Clin Oncol (R Coll Radiol). 2023 Jun;35(6):397–407. doi: 10.1016/j.clon.2023.03.007 (PMC10186116; doi:10.1016/j.clon.2023.03.007)
Supplement: Multimedia component 3 [file mmc3.docx]

# Supplementary Appendix 3: Treatments and Baseline Characteristics of Patients Included in the SECRAB Quality of Life Sub-study that were Comparable to the Parent Trial

|  | **Quality of Life Sub-study** | | |
| --- | --- | --- | --- |
|  | **Synchronous** | **Sequential** | **Total** |
|  | N=291 (%) | N=280 (%) | N=571 (%) |
| **Age (years)** |  |  |  |
| Median | 52 | 51 | 51 |
| Interquartile range | 44 – 58 | 46 – 58 | 44 - 58 |
| <50 | 121 (42) | 121 (43) | 242 (42) |
| >50 | 170 (58) | 159 (57) | 329 (58) |
| **Chemotherapy Regimen Intent** | |  |  |
| CMF | 144 (49) | 139 (50) | 283 (50) |
| Anthracycline-CMF | 144 (49) | 139 (50) | 283 (50) |
| MMM | 3 (1) | 2 (1) | 5 (1) |
| **RT Schedule Intent** |  |  |  |
| 3-weekly | 201 (69) | 186 (66) | 387 (68) |
| >3-weekly | 88 (30) | 94 (34) | 182 (34) |
| Unknown | 2 (1) | - | 2 (0) |
| **Type of Surgery** |  |  |  |
| Mastectomy | 145 (50) | 137 (49) | 280 (49) |
| Wide Local Excision | 145 (50) | 141 (50) | 286 (50) |
| Other | 1 (0) | 2 (1) | 3 (1) |
| **Vascular/Lymphatic Invasion** | |  |  |
| Not seen | 151 (52) | 147 (53) | 298 (52) |
| Present | 138 (47) | 132 (47) | 270 (47) |
| Unknown | 2 (1) | 1 (0) | 3 (1) |
| **Number of Nodes** |  |  |  |
| Negative | 177 (40) | 114 (41) | 231 (40) |
| 1 – 3 positive | 114 (39) | 93 (33) | 207 (36) |
| 4+ positive | 60 (21) | 73 (26) | 133 (23) |
| **Tumour Grade** |  |  |  |
| Grade 1 – Well differentiated | 21 (7) | 13 (5) | 34 (6) |
| Grade 2 – Moderately differentiated | 102 (35) | 97 (35) | 199 (35) |
| Grade 3 – Poorly differentiated | 162 (57) | 168 (60) | 333 (58) |
| Unknown | 3 (1) | 2 (1) | 5 (1) |
| **Tumour Size (mm)** |  |  |  |
| N | 289 | 276 | 565 |
| Median | 22 | 23 | 22 |
| Interquartile range | 16 – 29 | 16 - 30 | 16 - 30 |
| Range | 5 – 85 | 2 – 170 | 2 – 170 |
| **ER Status** |  |  |  |
| Negative | 91 (31) | 92 (33) | 183 (32) |
| Positive | 186 (63) | 177 (63) | 363 (63) |
| Unknown | 14 (5) | 11 (4) | 25 (4) |
| **PgR Status** |  |  |  |
| Negative | 56 (19) | 64 (23) | 120 (21) |
| Positive | 78 (27) | 65 (23) | 143 (26) |
| Unknown | 157 (54) | 151 (54) | 308 (54) |
| **HER2 Status** |  |  |  |
| Negative | 30 (10) | 28 (10) | 58 (10) |
| Positive | 14 (5) | 9 (3) | 23 (4) |
| Unknown | 247 (85) | 243 (87) | 490 (86) |
| **Present Menopausal Status** |  |  |  |
| Pre | 100 (34) | 103 (37) | 203 (36) |
| Peri | 23 (8) | 25 (9) | 48 (8) |
| Post | 138 (47) | 128 (46) | 2626 (47) |
| Unknown | 30 (10) | 24 (9) | 54 (9) |
| **Other Hormone Manipulation** | |  |  |
| No | 286 (98) | 275 (98) | 561 (98) |
| Yes | 4 (1) | 3 (1) | 7 (1) |
| Unknown | 9 (0) | 2 (1) | 3 (1) |

CMF, Cyclophosphamide, methotrexate, 5-fluorouracil; ER, oestrogen receptor; HER2, Human Epidermal Growth Factor Receptor 2; MMM, Mitomycin-C, mitoxantrone and methotrexate; PgR, progesterone receptor.

Note: Percentages may not total 100 due to rounding.
