# Supplementary material for: Detailed Sub-study Analysis of the SECRAB Trial: Quality of Life, Cosmesis and Chemotherapy Dose Intensity
Source: Clin Oncol (R Coll Radiol). 2023 Jun;35(6):397–407. doi: 10.1016/j.clon.2023.03.007 (PMC10186116; doi:10.1016/j.clon.2023.03.007)
Supplement: Multimedia component 4 [file mmc4.docx]

# Supplementary Appendix 4: Multilevel Mixed-effects Modelling for the SECRAB Quality of Life Sub-study

| **Questionnaire / Measure*** | **Coefficient** | **95% CI** | **P-value** |
| --- | --- | --- | --- |
| **EORTC QLQ-C30** |  |  |  |
| Global health status | -0.05 | -2.16 – 2.06 | 0.963 |
| Physical functioning | 0.24 | 0.03 – 1.68 | 0.833 |
| Role functioning | -0.13 | -3.14 – 2.88 | 0.933 |
| Emotional functioning | -0.03 | -2.38 – 2.31 | 0.979 |
| Cognitive functioning | 0.02 | -2.19 – 2.22 | 0.989 |
| Social functioning | -0.15 | -2.93 – 2.63 | 0.917 |
| Fatigue | 0.04 | -2.38 – 2.46 | 0.974 |
| Nausea and vomiting | 0.03 | -1.75 – 1.82 | 0.973 |
| Pain | 0.15 | -2.16 – 2.46 | 0.898 |
| Dyspnoea | 0.23 | -2.43 – 2.88 | 0.867 |
| Insomnia | -0.05 | -3.58 – 3.48 | 0.978 |
| Appetite loss | -0.53 | -3.18 – 2.12 | 0.696 |
| Constipation | 0.22 | -2.34 – 2.78 | 0.868 |
| Diarrhoea | 0.03 | -1.75 – 1.82 | 0.973 |
| Financial difficulties | 0.21 | -2.60 – 3.02 | 0.884 |
| **EORTC QLQ-BR23** |  |  |  |
| Body image | -0.12 | -2.53 – 2.30 | 0.924 |
| Sexual functioning | -0.01 | -2.23 – 2.21 | 0.992 |
| Sexual enjoyment | -0.02 | -3.80 – 3.77 | 0.993 |
| Breast symptoms | 0.11 | -1.88 – 2.10 | 0.915 |
| Arm symptoms | -0.02 | -2.06 – 2.02 | 0.985 |
| Future perspectives | -0.12 | -3.29 – 3.04 | 0.939 |
| Systemic therapy side effects | 0.05 | -1.75 – 1.84 | 0.959 |
| Upset by hair loss | 0.26 | -8.18 – 8.70 | 0.952 |
| **Women’s Health Questionnaire** |  |  |  |
| Anxiety/fears | 0.04 | -3.17 – 3.25 | 0.980 |
| Attractiveness | -0.02 | -4.09 – 4.06 | 0.993 |
| Somatic symptoms | -0.03 | -2.45 – 2.38 | 0.979 |
| Memory/concentration | 0.04 | -3.74 – 3.83 | 0.982 |
| Vasomotor symptoms | -0.22 | -4.67 – 4.23 | 0.924 |
| Depressed mood | 0.03 | -2.70 – 2.76 | 0.983 |
| Sleep problems | -0.05 | -3.85 – 3.76 | 0.980 |
| Sexual behaviour | 0.02 | -4.37 – 4.40 | 0.994 |
| Menstrual symptoms | 0.01 | -2.75 – 2.76 | 0.997 |

* All three questionnaires were analysed using multi-level mixed effects models, where repeated measurements from baseline through to two-years post-surgery were analysed as random effects with stratification factors and radiotherapy schedule forced in to the model as fixed effects.
