# Supplementary material for: Detailed Sub-study Analysis of the SECRAB Trial: Quality of Life, Cosmesis and Chemotherapy Dose Intensity
Source: Clin Oncol (R Coll Radiol). 2023 Jun;35(6):397–407. doi: 10.1016/j.clon.2023.03.007 (PMC10186116; doi:10.1016/j.clon.2023.03.007)

# Supplementary Appendix 5: EORTC QLQ-BR23 Sub-scales Differences within the SECRAB Quality of Life Sub-study

The unadjusted mean and standard deviation of changes within the EORTC QLQ-B23 sub-scales in patients receiving synchronous or sequential chemo-radiotherapy are shown.

Questionnaire numbers: 1 - Baseline assessment prior to chemotherapy; 2 - on completion of both chemotherapy and radiotherapy; 3 – one-year following surgery; 4 – two-years following surgery.


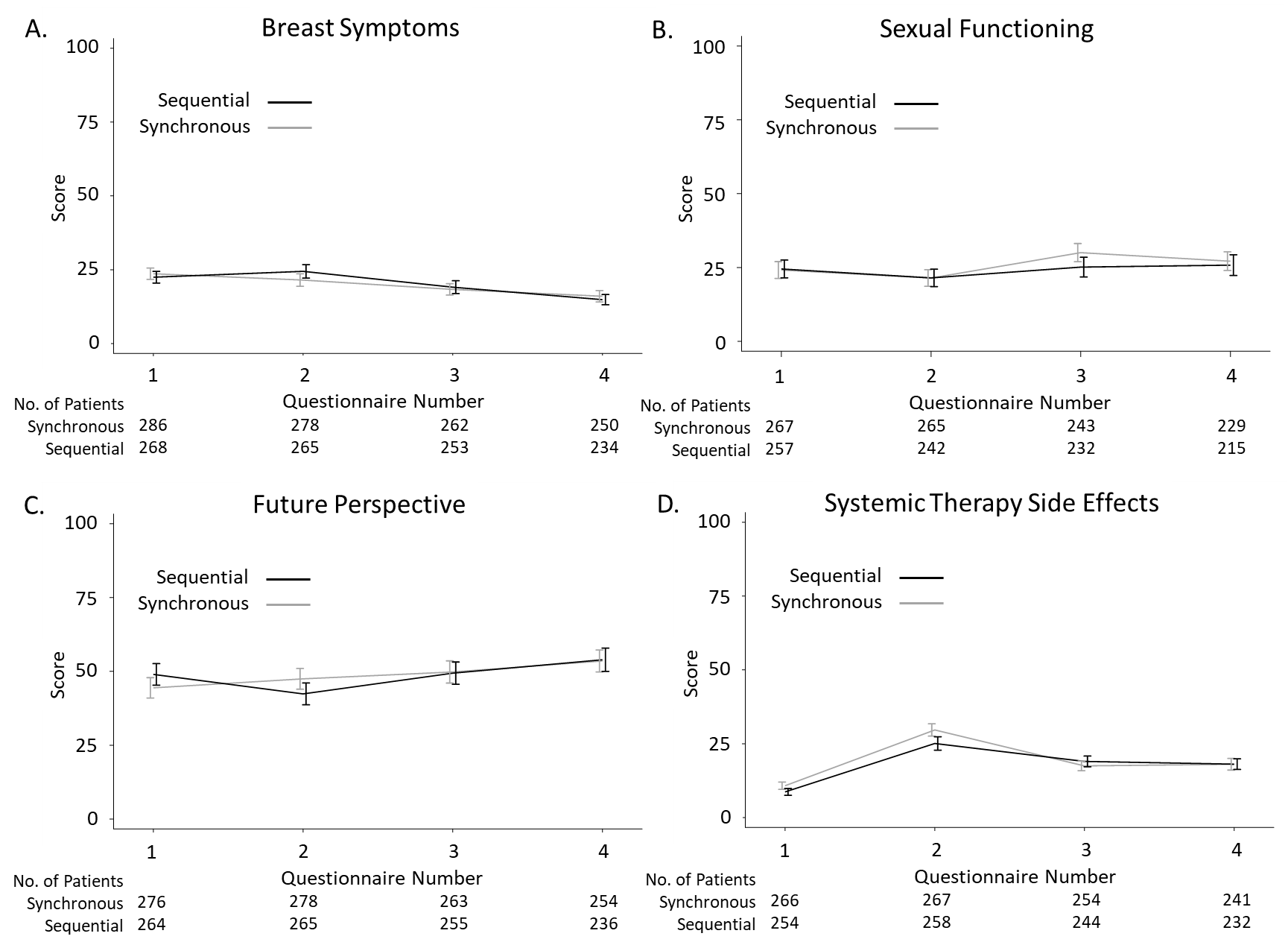

Supplement: Multimedia component 5 [file mmc5.docx]
