# Supplementary material for: Detailed Sub-study Analysis of the SECRAB Trial: Quality of Life, Cosmesis and Chemotherapy Dose Intensity
Source: Clin Oncol (R Coll Radiol). 2023 Jun;35(6):397–407. doi: 10.1016/j.clon.2023.03.007 (PMC10186116; doi:10.1016/j.clon.2023.03.007)
Supplement: Multimedia component 6 [file mmc6.docx]

# Supplementary Appendix 6: Treatments and Baseline Characteristics of Patients Included in the SECRAB Cosmesis Sub-study that were Comparable to the Parent Trial

|  | **Cosmesis Sub-study** | | |
| --- | --- | --- | --- |
|  | **Synchronous** | **Sequential** | **Total** |
|  | N=180 (%) | N=144 (%) | N=324 (%) |
| **Age (years)** |  |  |  |
| Median | 52 | 50 | 51 |
| Interquartile range | 45 – 58 | 44 – 57 | 44 - 58 |
| <50 | 78 (43) | 74 (51) | 152 (47) |
| >50 | 102 (57) | 70 (49) | 172 (53) |
| **Type of Surgery** |  |  |  |
| Mastectomy | 93 (52) | 65 (45) | 158 (49) |
| Wide Local Excision | 85 (47) | 77 (53) | 162 (50) |
| Other | 2 (1) | 2 (1) | 4 (1) |
| **Vascular/Lymphatic Invasion** | |  |  |
| Not seen | 93 (52) | 77 (53) | 170 (52) |
| Present | 86 (48) | 67 (47) | 153 (47) |
| Unknown | 1 (0) | - | 1 (0) |
| **Number of Nodes** |  |  |  |
| Negative | 74 (41) | 69 (48) | 143 (44) |
| 1 – 3 positive | 69 (38) | 51 (35) | 120 (37) |
| 4+ positive | 37 (21) | 24 (17) | 61 (19) |
| **Tumour Grade** |  |  |  |
| Grade 1 – Well differentiated | 12 (7) | 3 (2) | 15 (5) |
| Grade 2 – Moderately differentiated | 61 (34) | 52 (36) | 113 (35) |
| Grade 3 – Poorly differentiated | 106 (59) | 89 (62) | 195 (60) |
| Unknown | 1 (1) | - | 1 (0) |
| **Tumour Size (mm)** |  |  |  |
| N | 179 | 142 | 321 |
| Median | 22 | 22 | 22 |
| Interquartile range | 17 – 28 | 17 – 30 | 17 – 29 |
| Range | 5 – 85 | 6 – 100 | 5 – 100 |
| **Present Menopausal Status** |  |  |  |
| Pre | 58 (32) | 57 (40) | 115 (35) |
| Peri | 11 (6) | 12 (8) | 23 (7) |
| Post | 92 (51) | 60 (42) | 152 (47) |
| Unknown | 19 (11) | 15 (10) | 34 (10) |
| **Other Hormone Manipulation** | |  |  |
| No | 175 (97) | 140 (97) | 315 (97) |
| Yes | 4 (2) | 4 (3) | 8 (2) |
| Unknown | 1 (1) | - | 1 (0) |

Note: Percentages may not total 100 due to rounding.
