# Supplementary material for: Detailed Sub-study Analysis of the SECRAB Trial: Quality of Life, Cosmesis and Chemotherapy Dose Intensity
Source: Clin Oncol (R Coll Radiol). 2023 Jun;35(6):397–407. doi: 10.1016/j.clon.2023.03.007 (PMC10186116; doi:10.1016/j.clon.2023.03.007)
Supplement: Multimedia component 7 [file mmc7.docx]

# Supplementary Appendix 7: SECRAB Trial Steering Committee

At the request of the Editors at Clinical Oncology, this appendix will be including following blind-review.
